# Shelf life of sterilized packed items stored in CSSD of a Vietnam University Medical Center

**DOI:** 10.1017/ash.2025.104

**Published:** 2025-09-03

**Authors:** Nguyen Vu Hoang Yen, Vu Thi Cham, Trinh Thi Thoa, Pham Thi Thuy, Duong Thi Tam, Hoang Kim Ngan, Lu Thi Mong Huong, Thai Hong Van, Nguyen Thi Mong Huyen, Tran Hoang Thanh, Nguyen Duc Duy, Nguyen Do Phuong Thao, Le Vo Hong Tuyet, Huynh Minh Tuan

**Affiliations:** 1 University Medical Center, Ho Chi Minh City, Viet Nam; 2 University of Medicine and Pharmacy at Ho Chi Minh City

## Abstract

**Background:** In University Medical Center Ho Chi Minh City (UMC), shelf life of sterilized packed items has been followed by time-related principle. However, duration of sterility has not been based on strong scientific evidence. **Objectives:** To determine the most appropriate shelf life for sterilized products according to packaging material and sterilization methods. **Methods:** All the experimental and the control samples (surgical instruments and linen) were prepared by four types of packaging materials (peel pouch, nonwoven, linen, and rigid container) and three types of sterilization methods (steam, Hydrogen Peroxide, Ethylene Oxide). After sterilization, sterilized samples were stored at CSSD’s storage and tested for microbial contamination in 07 periods: after 07 days, 14 days, 01 month, 03 months, 06 months, 12 months, and 18 months. Identification of the storage environment (shelf location, temperature, and relative humidity) were recorded as the same time collected samples. **Results:** Positive microbial cultures were seen in 0.44% (07 samples) of 1,574 samples. Up to 18 months, no organisms was cultured from any sample of (1) autoclaved surgical instrument packages wrapped in peel pouches, nonwoven, linen, (2) Hydrogen Peroxide sterilized surgical instrument packages wrapped in nonwoven, (3) Ethylene Oxide sterilized surgical instrument packages, and (4) autoclaved linen packages wrapped in nonwoven. Organisms detected were both Gram–Positive and Gram-Negative bacteria. Just only approximately 17% control samples grew bacteria. There was no any statistically significant relationship between positive experimental samples and packaging materials, sterilization methods, or storage conditions. **Conclusions:** Based on results of this experiment, shelf life of sterilized packed items should be still followed by time-related principle in UMC. However, the currently shelf life can be extended to reduce unnecessary costs and increase the usage rotation.

